# The Effects of Processing Non-Timber Forest Products and Trade Partnerships on People's Well-Being and Forest Conservation in Amazonian Societies

**DOI:** 10.1371/journal.pone.0043055

**Published:** 2012-08-17

**Authors:** Carla Morsello, Isabel Ruiz-Mallén, Maria Dolores Montoya Diaz, Victoria Reyes-García

**Affiliations:** 1 Escola de Artes, Ciencias e Humanidades (EACH), Universidade de São Paulo, São Paulo, São Paulo, Brazil; 2 Institut de Ciència i Tecnologia Ambientals, Universitat Autònoma de Barcelona, Bellaterra, Barcelona, Spain; 3 Faculdade de Economia, Administração e Contabilidade, Universidade de São Paulo, São Paulo, São Paulo, Brazil; 4 ICREA and Institut de Ciència i Tecnologia Ambientals, Universitat Autònoma de Barcelona, Bellaterra, Barcelona, Spain; University of Marburg, Germany

## Abstract

This study evaluated whether processing non-timber forest products (NTFPs) and establishing trade partnerships between forest communities and companies enhance the outcomes of NTFP commercialization. In particular, we evaluated whether product processing, partnerships, or their combination was associated with a number of outcomes related to the well-being of forest inhabitants and forest conservation. We based our analyses on ethnographic and quantitative data (i.e., survey and systematic observations) gathered at seven communities from five societies of the Brazilian and Bolivian Amazon. Our results indicated that product processing and partnerships do not represent a silver bullet able to improve the results of NTFP commercialization in terms of well-being and conservation indicators. Compared with cases without interventions, households adopting partnerships but not product processing were most often associated with improved economic proxies of well-being (total income, NTFP income, food consumption and gender equality in income). In comparison, the combination of product processing and partnerships was associated with similar outcomes. Unexpectedly, product processing alone was associated with negative outcomes in the economic indicators of well-being. All of the investigated strategies were associated with less time spent in social and cultural activities. With respect to forest conservation, the strategies that included a partnership with or without processing produced similar results: while household deforestation tended to decrease, the hunting impact increased. Processing alone was also associated with higher levels of hunting, though it did not reduce deforestation. Our results indicate that establishing partnerships may enhance the outcomes of NTFP trade in terms of the financial outcomes of local communities, but practitioners need to use caution when adopting the processing strategy and they need to evaluate potential negative results for indicators of social and cultural activities. With respect to conservation, the three strategies are promising for reducing deforestation, but more pervasive impacts, such as hunting, might increase.

## Introduction

Trade in non-timber forest products (NTFPs), such as fruits, nuts or fibers, was proposed in the 1990s as a strategy able to reconcile conservation and development goals in poor forest communities [1], [2], [3], [4]. Proponents have stressed the low environmental impact of NTFP extraction [5], the ability to prevent the conversion of forests to other land uses [4], the cultural appropriateness of the strategy [6], the low entry barriers [7], and the safety net function of NTFPs [8], [9]. Encouraged by the possibility of a win-win scenario, many indigenous and conservation advocacy groups have promoted markets for NTFPs in tropical forests [10].

Later on, however, evaluations of the pros and cons of trading NTFPs tempered the enthusiasm for this idea (for reviews, see [11], [12], [13], [14], [15]). On the development side, some studies, particularly those from humid tropical forests, showed that NTFPs are unable to alleviate poverty, although they may prevent poverty intensification [9], [11], [16]. In contrast, in drier woodlands and savannas, characterized by a markedly drier and variable climate which limits the returns from agriculture, NTFPs may significantly contribute to local livelihoods and even offer a pathway out of poverty [17]. Nevertheless, NTFP trade may also generate conflicts over resource use or inequalities because of differential access to resources or trade [12], [17], and can imperil other subsistence practices such as agriculture, when the calendar of extraction or processing overlaps with other tasks [18]. From a conservation perspective, the evaluations concluded that extraction may lead to landscape impacts because of trail opening and road building [19], cause overexploitation and problems for the species exploited and their dispersers or predators [20], and displace natural vegetation because of intensively managed NTFP production [13]. Finally, evidence showed the alleged conservation-development link may be misleading, as there is often a trade-off: better conservation outcomes are often associated with worse development outcomes and vice-versa [9], [19], [21].

Because of the disenchantment with NTFP trade at the turn of the century [15], the academic discussion has increasingly migrated towards approaches based on Payments for Environmental Services [22], or to more nuanced understanding and policy recommendations that emphasize the multiple use of forests and the adoption of strategies that increase the returns from NTFP commercialization [15], [23]. In this regard, from the beginning of the discussion about the NTFP potential, two strategies have been proposed to boost the outcomes of NTFP trade. The first strategy is to implement processing, storing, or packing at the community level [24]. Processing should add value to the production, reduce the urgency to sell and allow the collection of larger volumes of products, therefore enhancing the financial benefits for local people, improving their bargaining power with buyers, and reducing transportation costs [13], [25]. The second strategy is to establish partnerships between NTFP extractors and business companies, non-governmental organizations (NGOs) and other players that might aid in the production and commercialization processes. Such partnerships vary widely, but are often considered as more or less formal arrangements between two or more parties who share goals, responsibilities and risks in the expectation that each will gain from the arrangement [26]. Partnerships should enhance the local people's economic benefits because trade is expected to become more stable, companies would purchase directly from producers paying premium prices, and companies would aid in the improvement of technology, infrastructure and market access [27], [28]. Moreover, partnerships might also improve the conservation outcomes because partner companies target niche markets that demand social and environmental responsibility in production [29], [30].

Although these two strategies have often been cited as decisive in improving the outcomes of NTFP commercialization, we still lack rigorous and systematic evaluations of the consequences of partnerships and NTFP processing across diverse settings. Moreover, previous studies usually evaluate the effects considering the impacts at the community level, despite consequences may vary to different households, or are based on people's perceptions instead of direct observation of the effects. In this study, we contribute to filling this gap. Specifically, we use data from five Amazonian societies to test whether NTFP processing and the establishment of partnerships between companies and forest communities improve a number of outcomes related to (i) the well-being of forest inhabitants and (ii) forest conservation. We find that partnerships are associated with improved outcomes in economic and deforestation terms, but cultural and social aspects are negatively affected, and more pervasive impacts, such as hunting, increase. Product processing in our context is often associated with negative outcomes to well-being and conservation.

## Methods

### The Case Studies

Our analysis is based on data from seven communities that belong to five small-scale Amazonian societies. We focus on Amazonian cases because, since the 1990s, the region has experienced an expansion in company-community partnerships for NTFP trade, which span a variety of sectors (e.g., cosmetics and food) and products (e.g., essential oils and fibers) [29].

Data from three indigenous Brazilian communities (Kayapó from A'Ukre, Araweté from Igarapé Ipixuna, and Asuriní do Xingu from Koatinemo), and from two caboclo communities from the Médio Juruá Extractive Reserve (Roque and Pupuaí) originated from two projects aimed at evaluating company-community partnerships (http://www.parceriasflorestais.org). Data on two Tsimane' (Bolivia) communities (San Antonio and Yaranda) came from the Tsimane' Amazonian Panel Study (http://www.tsimane.org). Table 1 presents a summary of the societies and communities studied, organized according to the presence or absence of a partnership and NTFP processing.

**Table 1:** Description of Communities, NTFP Production, Product Processing and Partnership Status.

*Characteristics of societies studied*							
	[1]	[2]	[3]	[4]	[5]	[6]	[7]
Ethnic group	Tsimane'	Caboclo	Araweté	Asuriní do Xingu	Tsimane'	Kayapó	Caboclo
**Name of the community**	San Antonio	Pupuaí	Ipixuna	Koatinemo	Yaranda	A'Ukre	Roque
**Country (state)**	Bolivia (Beni)	Brazil (Amazonas)	Brazil (Pará)	Brazil (Pará)	Bolivia (Beni)	Brazil (Pará)	Brazil (Amazonas)
**Legal type of Territory**	Indigenous territory	Extractive Reserve	Indigenous Territory	Indigenous Territory	Indigenous Territory	Indigenous Territory	Extractive Reserve
**Language**	Tsimane'	Portuguese	Araweté	Asuriní	Tsimane'	Kayapó	Portuguese
**Community population**	170	186	326	122	164	263	480
**Number of households in the sample (% of total)**	35 (90%)	23 (100%)	30 (56%)	12 (100%)	31 (90%)	24 (100%)	25 (43%)
**Survey period**	22/04/2002 to 25/08/2003	23/04/2005 to 19/11/2005	06/01/2005 to 02/12/2005	03/01/2005 to 23/10/2005	22/04/2002 to 25/08/2003	14/05/1999 to 03/05/2000	23/04/2005 to 19/11/2005
**Data gathering periods**	5	2	3	3	5	4	2

*Characteristics of NTFP production and commercialization*							
**Presence of partnership**	No	No	Yes	Yes	No	Yes	Yes
**NTFP processing**	No	No	No	No	Yes	Yes	Yes
**% or number of households involved**	n.a.	n.a.	100%	88.8%	100%	100%	83.6%
**Partner company (sector)**	n.a.	n.a.	The Body Shop (Cosmetics)	The Body Shop (Cosmetics)	Independent traders (Construction)	The Body Shop (Cosmetics)	Natura/Cognis (Cosmetics)
**Main products commercialized**	Cajñere *(Ochroma pyramidale* Cav. ex Lam.*)* Urb.	Açaí *(Euterpe oleracea* Mart.*)*	Brazil nut (*Bertholettia excelsa* Humb & Bonpl.)	Brazil nut (*Bertholettia excelsa* Humb & Bonpl.)	Jatata (*Geonoma deversa*, (Poit.) Kunth); giant grass *Gynerium sagittatum* (Aubl.) P. Beauv. and *tapi* (*Thoracocarpus bissectus* (Vell.) Harling).	Brazil nut (*Bertholettia excelsa* Humb & Bonpl.)	Andiroba (*Carapa guianensis*, Aubl.); murumuru (*Astrocaryum murumuru*, Mart.)
**Plant part used**	Fruit	Fruit	Fruit (nuts)	Fruit (nuts)	Palm leaves	Fruit (nuts)	Fruits
**End use**	Mattresses	Food	Input for oil	Input for oil	Roofing mats	Cosmetic oil	Cosmetic oil

The seven communities share similar locations in Amazonian forests, prevailing modes of production and sources of income. All have remained highly autarkic and rely on similar subsistence practices, including a mix of hunting, gathering, fishing, and small-scale shifting agriculture. The communities also have access to similar sources of monetary income, including the commercialization of timber and NTFPs (e.g., mahogany and Brazil nuts) or handicrafts (e.g., arrows and ceramics), government subsidies, remittances, and wages from labor performed for outsiders (e.g., guides and work on the homestead of colonist farmers) or government institutions (e.g., teachers).

Despite similarities, the communities vary in their levels of exposure to markets, the language they speak, and cultural characteristics. Some of the indigenous groups have entered into contact with the surrounding society more recently (Araweté, Asuriní) or are found in remote locations (Araweté, Kayapó), whereas others have been exposed to the national societies for longer periods (Kayapó, Tsimane') or have more frequent interactions (Asuriní, some Tsimane').

Five of the communities belong to indigenous groups from Brazil (Araweté, Kayapó and Asuriní) and Bolivia (Tsimane'), whereas two are Caboclo communities. Caboclo societies comprise individuals who originated from mixed European, African and indigenous ancestors and have spent most, if not all, of their lives in forested regions. Hence, they have developed livelihoods and modes of resource use similar to those practiced by indigenous groups, despite speaking Portuguese and sharing cultural characteristics with the Brazilian society [31].

The seven communities present different types of arrangements for the production and commercialization of NTFPs, mainly with regard to product processing and the presence of a company-community partnership. We equate processing with the post-harvest transformation of NTFPs gathered into other products, such as the transformation of nuts into vegetable oils or palm leaves into roofing mats. With respect to the partnerships, our cases may be classified as productive partnerships or partnerships aimed at commercial production and trade [26]. All of the partnerships studied involve cosmetics companies, which is one of the leading sectors in these Amazonian agreements [29].

Two communities, the Tsimane' from San Antonio and the Caboclo from Pupuaí, do not heavily rely on NTFP trade. In these communities, people collect and occasionally trade a variety of minor NTFPs (see Table 1), but commercialization is not a main economic strategy, nor they have established a partnership with a company.

One community, the Tsimane' from Yaranda, relies heavily on processed NTFPs but has not established a partnership. Villagers themselves barter handmade roofing mats mainly made of palm leaves (Table 1) with visiting traders, or more sporadically in towns.

Between 1998 and 2009, two indigenous communities, the Asuriní and Araweté, relied on unprocessed NTFPs traded through a partnership with the multinational cosmetic company The Body Shop. Trade involved the commercialization of raw Brazil nuts (*Bertholletia excelsa* Humb. & Bonpl.), which were gathered by men and women [32], [33], and then traded through a cooperative controlled by government employees (Amazoncoop) working in 10 indigenous communities from 9 ethnic groups. In 2005, Brazil nuts were purchased at a price of US$12 a box (∼23 kg), which was over double the regional market price. The nuts were transformed into vegetable oil in a processing plant constructed in the town of Altamira and operated by urban-based indigenous peoples [33].

The Kayapó indigenous community and the Caboclos from Roque have signed agreements that involved product processing and trading with partner companies. The Kayapó pioneered indigenous trade partnerships in Brazil with The Body Shop. The deal, signed in 1991, consisted in trading a maximum of 2,000 kg of Brazil nut oil per year, sold at a premium price (i.e., US$35 per kg, or approximately four times the market price) [18]. The company initially managed all the duties and provided all necessary inputs, equipment and transportation, but this responsibility was later passed on to Amazoncoop. Kayapó men, women, and children gathered Brazil nuts that were then locally transformed into oil in a three-step processing cycle (shelling nuts, grinding, and pressing for oil extraction). Men and women shelled nuts, but only men performed the rest of the processing steps. People benefited financially from selling raw nuts (US$37/bag) or working by the hour in the shelling or oil processing (US$1–3/hour).

In 2000, the Caboclos from Roque established a partnership between their cooperative (CODAEMJ) and two companies: a multinational chemical company (Cognis) and the Brazilian cosmetics company (Natura). Caboclo men, women, and children gather two fruits: *andiroba* (*Carapa guianensis*, Aubl.) and *murumuru* (*Astrocaryum murumuru*, Mart.). The cooperative is responsible for purchasing the fruits and transporting them to Roque, where the fruits are processed into cosmetics oil through a semi-industrial process. *Murumuru* is hand shelled, predominantly by women, and oil is then extracted, whereas *andiroba* is only dried, and oil is later extracted by machine pressing of heated nuts. In both cases, filtering follows oil extraction. In a rotating duty cycle, three men each season operate the machinery for oil extraction. Cognis performs the final filtering and processing and resells the product to Natura, its only buyer. Cognis deals directly with the community, is responsible for capacity building, and eventually advances funds, while Natura establishes product demand and benefits from advertising the partnership [34]. Community members benefit financially from selling raw fruits (US$0.15/liter of *andiroba*; US$0.11/liter of *murumuru*), from working sporadically in the processing facility (i.e., shelling fruits and transportation), and from a few permanent jobs at the cooperative. In 2005, both oils were sold to Cognis at US$7 per liter.

At the locations where there was a partnership and NTFP processing in place, not all of the households in the communities benefited financially or invested their time in activities related to these agreements (Table 1).

### The Sample

The data used in the analyses combine qualitative ethnographic information collected through several months of fieldwork (from 7–16 months, depending on the location) with quantitative methods (specified below). We analyzed quantitative data at the household level because households are the units of economic organization in the studied societies. We defined a household as a group of people who share production (i.e., agriculture and hunting) and consumption (i.e., individuals who cook on the same fire) on a regular basis.

At each household, we gathered data from at least two and at maximum five different periods (Table 1). To standardize data collection periods across study sites, we classified them in the four more or less defined seasons in the region (i.e., dry season, colder station with wind, rainy season, transition dry/rainy season), which in turn are associated with the type of productive activities people engage on. Due to mobility and the repeated nature of data collection, we had to deal with temporary attrition and new arrivals. Since we collected data on arrival and departures at the individual level, we were able to define a common criterion of inclusion/exclusion of adults in the pooled sample, i.e., adults who stayed in the community for more than 60 days were included in the sample. The percentage of the total population from which we collected quantitative information varied across the communities (Table 1).

### The Models

The main aim of this study was to estimate the association between different types of arrangements for the commercialization of NTFPs with proxies of well-being and conservation. Specifically, we assessed the association between a set of dummy variables that captured the presence or absence of NTFP processing and/or trade partnerships (explanatory variables) and proxies of (i) well-being or (ii) conservation (dependent variables).

Because our main question referred to the effects of partnerships and product processing on well-being and conservation, our evaluation of these interventions should have established what would have happened to households in the absence of such interventions (the counterfactual) [35]. The best way to identify a causal relationship is to conduct a randomized experiment, but this procedure was unfeasible for ethical and budgetary reasons, and thus our study relied on observational data. A well-known problem of observational data is selectivity bias, that in our case could have occurred if, for instance, only households with some characteristics systematically adopted one type of intervention. If so, differences observed in the outcomes could be due to differences between treated and control groups in factors that may also have affected the outcomes, rather than from the interventions themselves [36]. To deal with selectivity bias, we used a pos-hoc method, propensity score analysis, because it would allow us to have a more rigorous evaluation of the conservation and development interventions (e.g., [37]).

The propensity score analysis reduces the bias in the estimation of the treatment effect, through balancing the distribution of covariates across treated and control groups [38]. The method creates the observational analogue of a social experiment in which everyone has the same probability of participation, thus ensuring that units are comparable and allowing the use of methods of analysis appropriate for randomized experiments [39]. To enhance comparability, data are preprocessed using propensity scores, which facilitates the construction of matching sets with similar distributions of covariates [38].

Specifically, we used propensity score weighting, a procedure that employs the propensity score to construct weights, which are then used to reweigh treatment and control groups to make them representative of the population of interest [40], [41]. This method was more appropriate than other propensity score methods because our treatment variable included four categories (i.e., without interventions, with partnership, with product processing, and with partnership and product processing). When the treatment can have different levels (e.g., different doses of a medicine), or different categories, as in our case, Imbens [42] has proposed a generalization of the classical, binary treatment propensity score to treatments with more than two categories. We followed Imbens [42] and calculated the propensity score with a multinomial model. Moreover, we chose a multinomial probit over a logit specification, because multinominal probit models allow one to relax the assumption of independence of irrelevant alternatives. Thus in our case the propensity score is a scalar of conditioning variables which determine the probability of a household receiving treatment [40].

We included in the model (i) variables unaffected by participation (or the anticipation of participation), and (ii) variables that simultaneously influenced the decision to participate in one of the interventions and the outcome variables [43]. We tested for a set of conditioning variables that were likely to be correlated with participation in the treatment groups and with the outcome variables. We included in the propensity score those variables that estimated average age of household people and average education of household adults, because both were unbalanced between control groups and treatments.

We then ran the regressions with the outcomes of well-being and conservation, while reweighting the sample by the inverse of the estimated propensity score [42]. We implemented a set of multilevel or mixed-model linear regressions, which are extensions of linear regressions appropriate for data with hierarchical structures, such as when sampled households are clustered within communities. These models were chosen because ignoring the hierarchical structure of data risked rendering invalid some traditional statistical analyses given that our units of analysis (households) could not be considered independent at the level of communities [44]. Because multilevel models produces unbiased and often more conservative standard errors, confidence intervals and significance tests [45], ignoring the structure could have increased the chance of finding significant relationships at the level of households.

With one exception, the models constructed are two-level random-intercept mixed-model linear regressions, in which level-1 households are nested within seven level-2 communities or villages. For the analysis of income irregularity, however, we used an Ordinary Least Squares regression because, in this case, we calculated a single measure per household for all quarters. In all these models the outcome variables are well-being and conservation indicators, while the explanatory variables are dummies for the presence or absence of partnerships and/or product processing. The regressions also included a set of controls for standard household covariates that might have affected the outcomes (i.e., household size, average household age, average adult education and a dummy to identify if the household head was a woman). We ran the statistical analyses in Stata® 2009 v.11.1.

Note that the sample size fluctuates across the regressions for three reasons. First, no data on consumption were collected for the Kayapó, so we had to exclude them when evaluating *food consumption* and *wild animal offtake*. Second, for the Tsimane', the variables constructed using weigh days (i.e., *food consumption* and *wild animal offtake*) or with spot sampling data (i.e., *leisure* and *hunting effort*) came from different years (1999 and 2002), so the sample size changes when using these variables. Third, some variables could not be measured for all households. For instance, inequality in income between women and men could not be calculated for households without adult men.

In the next sections, we define the variables used in the regressions and explain the hypotheses regarding the expected direction of the relationships. Table 2 and 3 present the definition of and summary statistics for the explanatory and outcome variables used in the regressions.

**Table 2:** Definition and Summary Statistics of Explanatory Variables Used in Regression Analyses (n = 180 households, and multiple observations per household).

Independent variables	Definition	Obs	#	%
Without partnership and without transformation (excluded category)	% of households in the category.	604	252	41.72
With partnership and without transformation	% of households in the category.	604	139	23.01
Without partnership and with transformation	% of households in the category.	604	114	18.87
With partnerships and with transformation	% of households in the category.	604	99	16.39

**Table 3:** Definition and Summary Statistics of Outcome Variables and Controls Used in Regression Analyses (n = 180 households, and multiple observations per household).

Variables	Definition					
A. Outcome variables		Obs	Mean	S. D.	Min	Max
*I. Well-being*						
1. Total income	Inputted monthly monetary and in-kind income earned by the household from barter, sales, remittances, wages, and gifts. In international dollars (PPP adjusted).	604	275.76	346.73	0	2,971.99
2. NTFP income	Inputted monthly monetary income earned by the household from the barter, sale, processing or working on managerial duties related to NTFP trade. In international US dollars (PPP adjusted).	604	48.20	149.21	0	1,718.65
3. Income irregularity	Ratio between the standard deviation and the mean of household income multiplied by a 100 (i.e., the coefficient of variation expressed in percentages)	125	146.35	38.83	0	233.54
4. Food consumption	Logarithm of the estimated monthly consumption of food by the household. In international dollars (PPP adjusted).	363	3.90	1.59	0	7.36
5. Gender equality	Z-score of the difference between the average income of adult women and adult men from the household at each quarter	578	0.03	1.11	−3.04	7.70
6. Leisure	Percentage of the total time budget spent on leisure (resting, playing, chatting, personal care, eating, drinking and ritual activities) by household adults in the quarter.	479	0.40	0.19	0	1
*II. Conservation*						
1. Deforestation	Total area cleared by a household in a year for agricultural plots. In square meters.	590	7,812.65	5,732.95	0	36,456.84
2. Wild animal offtake	Logarithm of the kilogram of hunted meat entering the household in a quarter, adjusted for one month.	363	0.90	1.61	0	6.25
3. Hunting effort	Percentage of the total time budget observations spent on hunting by adult men in the quarter.	241	0.04	0.08	0	0.5

B. Controls						
1. Household size	Number of people in the household in the quarter.	604	7.00	3.69	1	27
2. Household age	Average age of adults in the household in the quarter.	604	26.90	11.66	9.99	75.89
3. Education	Average level of education of household adults.	604	0.55	0.48	0	2
4. Woman household head	Household head is a woman (Yes = 1 or 2.32% of total, No = 0)	604	0.02	0.15	0	1

### Explanatory Variables: Product Processing and Partnership

We constructed four dummy variables to capture different situations regarding the production and commercialization of NTFPs. The variable omitted in the regressions, *without processing and without partnership*, was coded as 1 for households that neither benefit financially from processing the main product they gather, nor participate in a partnership with a company, and as 0 otherwise. The three other dummy explanatory variables, which were coded as 1 if applicable and 0 otherwise, include: (i) *with processing and without partnership*; (ii) *without processing and with partnership*; and (iii) *with processing and with partnership*. Remember also that, in the regressions, the sample was reweighted by the inverse of the estimated propensity score.

### Outcome Variables and Hypotheses Related to Well-being

To evaluate the effects of different production and commercialization strategies on the well-being of forest people, we used six proxies of household well-being, including both economic (i.e., total income, NTFP income, income irregularity, food consumption, and gender equality) and non-economic (i.e., leisure time) attributes. We acknowledge that our indicators do not include the entire possible spectrum of well-being and focus mainly on economic aspects, but there are two reasons for this. NTFP commercialization has been implemented mainly to improve economic standards of living [2], and therefore evaluation of these outcomes is relevant. Additionally, the link between NTFP trade and some indicators of well-being (e.g., health) is not evident, so we focused only on those indicators more directly linked to NTFP trade and the interventions considered in this study.

The first obvious indicator of economic well-being is income, because higher levels of income are often associated with objective levels of well-being, although there is also evidence that the marginal utility of income is low as income gets higher [46]. In our case, income was represented by *total income* and *NTFP income*. *Total income* refers to the sum of all of the income received from bartering, sales, remittances, wages and gifts, while *NTFP income* exclusively includes the amount of income derived from bartering, sales, processing or work involved in managerial duties related to NTFP trade. The data used to compute income variables originated from household surveys repeated quarterly (from 2 to 5 times) at each location. We asked each individual to report their sources of income during the 15 or 30 days prior to the interview (depending on the site) and then listed potential sources of income (i.e., wages, sales, remittances, bartering and gifts). Both income estimates are the sum (at the household level and for each quarter) of the specific income type adjusted to a monthly value. Values in local currencies were converted to international dollars using the annual index of Purchasing Power Parities provided by The World Bank (see http://data.worldbank.org).

As it has been argued that product processing and partnerships should increase people's access to income [27], [28], we expected the coefficients of the three explanatory variables that include the interventions (processing, partnership or both) to be positively correlated with *total income* and *NTFP income*. Product processing, such as the pos-harvesting transformation into oils, mats or other products, should increase NTFP income because processing aggregates value to the products commercialized. Moreover, processing opens up new opportunities for raising monetary income, which can be pursued in periods of the year when there are fewer economic options [47], therefore also increasing the levels of total income. Likewise, partnerships with companies should also lift the levels of NTFP income and total income, because directly trading with companies without intermediaries should boost the share of the total trade value received by local people [24]. Additionally, partnerships with companies engaged in fair trade markets are often accounted as paying premium prices to local communities [18], so their presence should increase NTFP income and total income.

Another indicator of economic well-being is income regularity, because having irregular sources can increase the risk to local people of facing periods of scarcity. In this study, *income irregularity (*i.e., fluctuations in income levels) was defined as the coefficient of variation, or the ratio between the standard deviation and the mean of the household income multiplied by 100, and expressed as a percentage. Higher values thus indicate that incomes are more irregular. Data to construct the variable came from the income data already defined, considering the variation among the periods of income estimates in one year.

*Income irregularity* was evaluated because researchers have argued that product processing and the presence of a partnership could smooth fluctuations in income levels [13], [25]. If so, we should find a negative association between *income irregularity* and the explanatory variables that include processing, a partnership or both. Product processing should smooth fluctuations in income levels across the year, because processing opens up new opportunities in periods of the year when there are few other economic options [47]. Likewise, partnerships should stabilize incomes because partner companies tend to guarantee product purchase [29] of otherwise highly unstable NTFP markets [17].

*Food consumption* is another more direct indicator of well-being which is not necessarily correlated with monetary income, since a great part of food consumption in remote rural locations is based on local production and gathering. We defined *food consumption* as the value (in international dollars) of the amount of food (i.e., locally produced and purchased) consumed in a month by the household. Data on *food consumption* came from weigh days, or the monitoring of products entering households at daylight on days chosen at random (see [48]). On those days, we counted, measured, and weighed all of the items entering the sampled houses to estimate the quantity of goods and their origin (e.g., market, forest, river). We then converted the goods consumed into their local currency equivalent, which was later transformed into international dollars. Because the number of observation days per quarter varied across locations (from 4–6), we averaged the daily observations for each quarter and adjusted them to a monthly value. We log-transformed this variable for ease of interpretation, and added the value one to avoid losing observations.

*Food consumption* was evaluated because there are contradictory arguments as to whether initiatives that increase households' integration into the market economy contribute to improve food consumption. On the one hand, because product processing and partnerships are likely to increase the access and the levels of monetary income, they may allow people to incorporate new products from the market and purchase food in times of scarcity, such as happens when Amazonian indigenous groups are integrated into markets [49]. On the other hand, partnerships and particularly product processing should take up people's time, so people may experience difficulties in producing and gathering local food if there are no opportunities for hiring labor [18]. In turn, more reliance on purchased food can increase food insecurity, because people may run out of food before having the cash to purchase more supplies [50]. Because in very remote contexts, such as ours, purchasing food is difficult, we expected the coefficients of the variables that include either a partnership or processing to be negatively correlated with *food consumption*.

*Gender equality* in income is another indicator of well-being, because household income can be raised without distributing it equally. Moreover, there is evidence that women's income contribute more to the well-being of the family than men's income [51]. We therefore checked for an association between the interventions (i.e., product processing, partnership, or both) and gender equality in income. Specifically, we calculated the difference between the average income of adult women and adult men at the household level for each period of data gathering. This value was then transformed into z-scores for ease of interpretation. Positive and larger values in the index indicate a more equal distribution of income between women and men. Note, however, that this indicator has limitations. The measure is mainly an economic index (in line with [52] and [53]), but it does not consider whether power relations between women and men are affected, nor if women's participate more in decision making or have control over the income generated (such as for example, the more qualitative measures of empowerment used in [54], [19], and [55]).

*Gender equality* was evaluated because there is mixed evidence regarding whether NTFP trade at large is associated with the empowerment [47], [53] or disempowerment [55] of women. Product processing has been reported to open up opportunities for cash earning among women who frequently dominate the processing phase [47], although there is also evidence of men taking over control of processing and income, even in activities previously run by women [11]. In our context, however, we expected processing to be positively associated with increases in gender equality, because there are very few opportunities for cash earning among women in the Amazonian context. As regards partnerships, there are also reports of gender inequality smoothing following trade agreements [56] because, allegedly, companies tend to promote more equal opportunities. We therefore expected that the three explanatory variables which included the presence of processing, partnership or both would be positively correlated with *gender equality*.

Our next indicator, *leisure*, is based on the assumption popularized by the seminal work of Sahlins [57] that the quantity of leisure time, on its own, represents well-being in particular contexts. Leisure is important because how people spend their time affects subjective perceptions of well-being, which do not necessarily correlate with objective indicators of well-being [58]. We equated *leisure* with the average percentage of time (i.e., total number of direct and reported observations) that adults were observed to be engaged in resting, playing, chatting, personal care, eating, drinking and ritual activities. To estimate *leisure*, we conducted scans or spot observations, also referred to as random-interval instantaneous sampling [59]. Scans were conducted during daylight in consecutive quarters, so our measure captures seasonal variations in time allocation. Following standard practice [59], we noted what subjects were doing when we first spotted them according to a pre-coded list. If the person was not present, we asked a proxy respondent (i.e., a relative from the same household) the whereabouts of the missed person. Whenever possible, we also checked later on with the own person the veracity of the information to increase the reliability of our estimates. Although asking people reduces the reliability of the measure, it was necessary to include this feature because several activities occurred outside of the village, so it was difficult or even impossible to observe them directly.

The evaluation of *leisure* is important in the present context because projects aimed at increasing monetary income may have the unintended consequence of reducing the time invested in social interactions and, thus, imperiling social bonds, which may themselves contribute to well-being [34]. Because product processing and dealing with partner companies (e.g., participation in meetings, managerial duties) require people's time investment, we expected the explanatory variables that include product processing and/or a partnership to be negatively associated with *leisure*. Note also that, although we have named the variable *leisure* for easiness, the indicator includes multiple forms of social interactions, including traditional festivals and rituals.

### Outcome Variables and Hypotheses Related to Conservation

Our estimates of conservation are indirect proxies that capture changes in natural resource use when people commercialize NTFPs: deforestation and hunting impact (measured here through two variables, *wild animal offtake* and *hunting effort*). We focused on indirect impacts because direct ecological impacts of extracting vegetable NTFPs depend on the part of the plant extracted, the rate of extraction and the local ecological conditions [60], so it was impossible to compare the impacts on the various resources extracted in our case studies due to their variability. Furthermore, indirect impacts are considered crucial for evaluating the results of NTFP commercialization, because the underlying logic that made NTFP trading popular was that the strategy should divert people from other activities that produce higher environmental impacts [19]. In particular, if NTFP trading promoted changes in livelihood practices that were associated with less deforestation and less hunting, forests would be better conserved.

To estimate *deforestation*, we calculated the total area, in square meters, cleared by a household for agricultural production in the survey year. At the Tsimane' villages, we used self-reports because previous research showed that the Tsimane' were able to accurately estimate the size of their agricultural plots [61] At the other sites, the area of each plot was directly measured with a hip chain and a compass.

*Wild animal offtake* and *hunting effort* are often used as proxies of hunting impact (e.g., [62]). For *wild animal offtake*, data were obtained from weigh days, as reported for the *food consumption* variable. The variable included in the regressions is the sum in kilograms of wildlife meat from different sources entering the household in a sample of days in each quarter. We log-transformed this variable for ease of interpretation, and one was added to avoid losing observations. For *hunting effort*, data came from spot checks, as explained for the variable *leisure*. People were coded as hunting when they were out with the intended purpose of hunting. The variable included in the regressions was the average of the percentage of the total number of observations in which adult men were reportedly hunting.

There is mixed evidence regarding whether partnerships and product processing should be associated with better or worse conservation outcomes. Partnerships and product processing may raise cash income, and there is evidence that cash transfers can increase the area of forests cleared for agriculture [63], [64], [65] or the demand for meat [66], [67], [68]. For instance, people may increase the area deforested because new cash sources promote the adoption of new technologies such as chainsaws [64], [69], or can increase hunting offtake if they purchase fire guns and ammunition more frequently [68]. However, increases in market exposure have also been associated with less deforestation [65], [70] and less hunting [71]. People may deforest less because, if they invest time in NTFP gathering and trade, they may lack the time to dedicate to agriculture [72]. Likewise they may diminish hunting effort for lack of time [64], or because they substitute hunted meat for purchased sources of protein [66]. Because processing and partnerships takes up people's time, we expected the independent variables that include product processing to be associated with less investment in agriculture and thus smaller areas cleared for agriculture, and less hunting (i.e., reduced offtake and hunting effort) because of less time investment and substitution with other products. Additionally, since partnerships are implemented to access niche markets that demand environmentally-friendly products [29], we expected them to be associated with better conservation outcomes, because companies allegedly promote strategies that reduce environmental impact. We therefore expected a negative association between our explanatory variables that included a partnership with *deforestation, wild animal offtake* and *hunting effort*.

### Ethics Statement

In Brazil, as demanded by the Brazilian law to do research in indigenous territories, our study protocol was evaluated by the Conselho Nacional de Desenvolvimento Científico e Tecnológico (CNPq), and then received approval by the Fundação Nacional do Índio (FUNAI 2/CGEP/04; 90/CGEP/04). The procedure also involved attesting researchers' health to avoid dissemination of contagious diseases. FUNAI was then responsible for obtaining verbal consent from the communities. To perform research at the Médio Juruá Extractive Reserve, we obtained written consent by the Instituto Nacional do Meio Ambiente e Recursos Naturais Renováveis (IBAMA; licence dated 16/01/2004), through the Centro Nacional para o Desenvolvimento Sustentado das Populações Tradicionais (CNPt). CNPt was then responsible for obtaining approval from the communities before releasing the written license. In Bolivia, the study protocol was approved by the Institutional Review Board for human subjects of Brandeis University (no number), Northwestern University (NUIRBS #1053-001) and the Great Tsimane' Council, responsible for obtaining approval at the community level.

In all the Brazilian and Bolivian locations, we began our fieldwork by organizing community meetings to explain the project objectives and methodology. We then obtained verbal consent from individuals that were willing to participate in the study. We did not ask for written consent at the individual level, since most people were illiterate and oral consent is more appropriate to the traditional practices of the small scale communities we studied.

## Results

We present the results in three sections. First, we ran a series of correlations between all our indicators of well-being and conservation to assess their level of overlap. We then present the regressions of the explanatory variables that include the presence or absence of product processing and partnerships with indicators of well-being. In the last section we repeat the analyses with indicators of conservation.

### Correlations Between Indicators of Well-being and Conservation

Before evaluating the association between our explanatory variables and the indicators of well-being and conservation, we had to understand whether these indicators captured different dimensions of those constructs. To do that, we ran a series of correlations of the indicators used as outcomes in the regression analyses (Table 4).

**Table 4:** Pairwise Correlation Coefficients for Well-being and Conservation Outcomes^a^.

	Well-being						Conservation		
	[1]	[2]	[3]	[4]	[5]	[6]	[7]	[8]	[9]
	Total income	NTFP income	Income irregularity	Food consumption	Gender equality	Leisure	Area deforested	Wild animals offtake	Hunting effort
**Well-being**									
a. Total income	1.000								
b. NTFP income	0.406***	1.000							
c. Income irregularity	−0.047	−0.0713	1.000						
d. Food consumption	0.136	0.133	−0.254***	1.000					
e. Gender equality	0.664***	0.132**	0.015	−0.110	1.000				
f. Leisure	−0.099	−0.0527	0.049	−0.170	−0.079	1.000			
**Conservation**									
g. Area deforested	0.018	−0.018	−0.048	0.248***	−0.090	0.023	1.000		
h. Wild animals offtake	0.307***	0.268***	0.006	0.432***	0.041	−0.220**	0.053	1.000	
i. Hunting effort	−0.124*	0.032	0.263**	−0.153	−0.111	−0.037	−0.037	0.072	1.000

**Notes:**.

(a) Šidák correction for multiple comparisons used.

As the results in Table 4 suggest, most of the selected indicators were not correlated. We only found coefficients larger than 0.5 and statistically significant at the 95% level in one out of 36 associations: total income and gender equality. Furthermore, only two other pairwise correlations approached the threshold: as expected, total income and NTFP income were consistently correlated; food consumption and wild animals offtake also displayed a statistical significant, but weaker, correlation. Note also that *total income* and *food consumption* were not highly correlated (column 1, row d), probably because the first captures monetary values well, but fails to represent the local production and gathering of products for own consumption.

### Well-being

Table 5 reports the regression results related to well-being. We present these results to focus attention on the different types of processing and partnerships (rows a, b, and c), and each type is compared with the excluded category (*without processing and without partnership*).

**Table 5:** Outcomes of Product Processing and Partnerships to Well-being.

	[1]	[2]	[3]	[4]	[5]	[6]
	Total income	NTFP income	Income irregularity	Food consumption (Log)	Gender equality	Leisure
**Independent variables** (excluded category is without processing and without partnership)	β∧ (S.E.)	β∧ (S.E.)	β∧ (S.E.)	β∧ (S.E.)	β∧ (S.E.)	β∧ (S.E.)
a. With processing and without partnership	−84.533 (31.105)***	−28.553 (6.253)***	41.205 (0.001)***	−0.328 (0.049)***	0.060 (0.056)	−0.104 (0.011)***
b. Without processing and with partnership	587.422 (90.389)***	163.972 (15.998)***	−16.816 (0.233)	2.184 (0.105)***	0.422 (0.168)**	−0.150 (0.030)***
c. With processing and with partnership	104.258 (8.650)***	77.731 (2.039)***	−1.601 (0.912)	0.601 (0.026)***	0.191 (0.068)***	−0.113 (0.027)***
**Controls**						
d. Household size	29.636 (12.372)**	11.849 (3.872)***	−0.515 (0.610)	0.059 (0.025)**	−0.019 (0.025)	−0.037 (0.024)
e. Household age	2.053 (0.882)**	1.048 (0.280)***	−0.079 (0.678)	−0.002 (0.009)	−0.007 (0.004)*	−0.003 (0.001)**
f. Household education	214.154 (90.625)**	30.777 (14.666)**	−22.441 (0.070)*	−0.372 (0.109)***	0.736 (0.202)***	−0.042 (0.044)
g. Woman household head	−38.770 (49.883)	−41.429 (18.954)**	30.459 (0.007)***	−0.373 (0.376)	0.507(0.051)***	−0.186 (0.128)
Constant	−71.712 (118.933)	−109.336 (42.525)**	177.620 (0.000)	3.099 (0.421)***	0.329 (0.325)	0.936 (0.301)***
**Random effects**						
*σ_u_* (a)	281.377 (114.471)***	72.615 (25.412)***	n.a(c)	1.085 (0.128)	0.352 (0.107)***	0.079 (0.021)***
*σ_ε_* (b)	232.013 (88.378)***	107.072 (41.382)***	n.a(c)	1.201 (0.120)*	1.119 (0.114)	0.362 (0.035)***
Intraclass correlation (Rho) or R^2^ in [3]	0.595	0.315	0.801(d)	0.449	0.090	0.045
**Observations**	604	604	125	365	578	479

**Notes:** Regressions are multilevel mixed-effects linear regressions, except [3] which is an OLS. All the regressions include robust standard errors and a full set of dummy variables for ethnic groups (not shown). Robust standard errors in parenthesis.

(a) Standard deviation (error term in parenthesis) at the community level (level 2);

(b) Standard deviation (error term in parenthesis) of the overall error term (household level 1);

(c) n.a = non applicable;

(d) Value refers to R^2^ in a Ordinary Least Square Regression.

*** p≤0.001;

** p≤0.05;

* p≤0.10.

Households involved in NTFP processing without the existence of a partnership (row a) were more often associated with negative measures of well-being than households in the excluded category. These associations were statistically significant for the variables total household income, NTFP income, income irregularity, food consumption and time spent on leisure. Compared with households *without processing and without partnership*, households with only processing but without partnerships on average (i) received US$84.53 less total monthly income (*p* = 0.007; column 1), (ii) received US$28.53 less NTFP monthly income (*p*≤0.001; column 2), (iii) had a more irregular income across the year (C.V. = 41.2%; *p* = 0.001; column 3), (iv) consumed 32.80% less food in dollars (*p*≤0.001; column 4) and (iv) spent approximately 1.0% less time on leisure (*p*≤0.001; column 6). Gender equality tended to increase (column 5), but the result was not statistically significant at the 10% level.

In contrast, households in the category *without processing and with partnership* (row b) were more frequently associated with positive outcomes for well-being than households in the excluded category. This statement holds and was statistically significant for total income, NTFP income, food consumption, and gender equality. Compared with the excluded category, households involved in a partnership but not in processing of NTFPs on average (i) received US$578.42 more total monthly income (*p*≤0.001; column 1), (ii) received US$163.97 more monthly NTFP income (*p*≤0.001; column 2), (iii) consumed 218.4% more food in dollars (*p*≤0.001; column 4), and (iv) the index of gender equality increased 42.2% points (*p = *0.012; column 5). In contrast, leisure time decreased by an average of 15.0% points (*p≤*0.001; column 6). Income irregularity tended to decrease, but this result was not statistically significant (*p* = 0.233).

The combination of product processing and the presence of a partnership was also more often associated with positive indicators of well-being (row c). In comparison with the excluded category, households involved in product processing within a partnership on average (i) received US$104.25 more total monthly income (*p*≤0.001; column 1), (ii) received US$77.73 more NTFP monthly income (*p*≤0.001; column 2), (iii) consumed 60.1% more food in dollars (*p*≤0.001; column 4) and (iv) exhibited a gender equality index 19.1% points higher than the excluded category (*p* = 0.005; column 5). Income irregularity tended to decrease, but this result was not consistent (*p* = 0.912). In contrast, involvement in product processing and a partnership was correlated with 11.3% less leisure time (*p*≤0.001; column 6).

### Conservation

Unlike the results regarding well-being, the associations concerning conservation (Table 6) varied less in relation to the type of NTFP intervention adopted. When compared to the excluded category, product processing, the presence of a partnership, or a combination of both mostly correlated in similar ways with the three indicators of conservation evaluated: deforested area, wild animal offtake and hunting effort. However, these results were opposite with respect to the two types of conservation outcomes evaluated: deforestation and hunting impact.

**Table 6:** Outcomes of Product Processing and Partnership to Forest Conservation.

	[1]	[2]	[3]
	Area deforested	Wild animals offtake	Hunting effort
**Independent variables** (excluded category is without processing and without partnership)	β∧ (S.E.)	β∧ (S.E.)	β∧ (S.E.)
a. With processing and without partnership	21.691 (459.762)	0.119 (0.085)	0.083 (0.006)***
b. Without processing and with partnership	−5,886.978 (1,042.668)***	3.527 (0.206)***	0.035 (0.014) **
c. With processing and with partnership	−7,427.650 (291.045)***	0.583 (0.010)***	0.031 (0.003)***
**Controls**			
d. Household size	450.171 (219.071)**	0.047 (0.042)	0.003 0.001)***
e. Household age	25.182 (11.820)**	−0.007 (0.005)	0.000 (0.000)
f. Household education	335.119 (1,534.251)	−0.088 (0.164)	−0.043 (0.025)*
g. Woman household head	128.666 (1,391.170)	−1.457 (0.465)***	−0.081 (0.003)***
Constant	8,889.800 (−2,579.728)***	0.322 (0.478)	−0.010 (0.015)
**Random effects**			
*σ_u_* (a)	3,237.092 (1,127.493)***	1.340 (0.534)	0.031 (0.012)***
*σ_ε_* (b)	2,883.014 (1,104.176)***	1.157 (0.091)*	0.080 (0.012)***
Intraclass correlation (Rho)	0.557	0.572	0.129
**Observations**	589	365	236

**Notes:** Regressions are multilevel mixed-effects linear regressions. All the regressions include robust standard errors and a full set of dummy variables for ethnic groups (not shown). Robust standard errors in parenthesis.

(a) Standard deviation (error term in parenthesis) at the community level (level 2);

(b) Standard deviation (error term in parenthesis) of the overall error term (household level 1).

*** p≤0.001;

** p≤0.05;

* p≤0.10.

In comparison to the excluded category, being engaged in a partnership and either processing or not the NTFPs gathered were associated with smaller average areas deforested areas by the households (column 1, rows b and c). These results were consistent and statistically significant for the interventions that included a partnership, but were in the opposite direction for the processing only strategy, though in this case the result was not statistically significant (row a). Specifically, when compared with the excluded category, households that were not engaged in processing but were involved in a partnership deforested an average of 5,886 m^2^ less (*p≤*0.001; row b), while households that processed NTFPs and were involved in a partnership deforested an average of 7,427 m^2^ less (*p≤*0.001; row c).

Opposite associations were observed with proxies for hunting, although the results were again consistent across the three explanatory variables. When compared to the excluded category, product processing, the existence of a partnership or a combination of both were generally associated with a greater hunting impact. Households that did not process NTFPs but benefited from a partnership harvested an average of 352.7% more kilograms of meat per month (*p≤*0.001; column 2, row b), while households benefiting from both product processing and a partnership harvested an average of 58.3% more (*p≤*0.001; column 2, row c). Households in the *with processing and without partnership* category also tended to harvest more meat in terms of kilograms than households in the excluded category, but the relationship was not statistically significant at the 10% level (column 2, row a). With respect to hunting effort, benefiting from a partnership, product processing or the combination of both were all significantly associated with average increases in hunting effort when compared with the baseline (column 3). The largest average increase in hunting effort (8.30% of the daily time schedule) was observed in those households that processed NTFPs but did not establish a trade partnership (*p≤*0.001; column 3, row a).

## Discussion and Conclusion

One finding stands out from our results: neither processing nor the existence of a partnership represented a silver bullet able to improve the results of NTFP trade with respect to all the well-being and conservation indicators evaluated.

Our data suggested that the best-case scenario regarding economic well-being were interventions based on trade partnerships with companies, without implementing NTFP processing at the community level. Partnerships alone displayed the best results in terms of total income, NTFP income, food consumption and gender equality. The observed benefits agree with prior studies proposing that company-community deals improve the financial returns of NTFP commercialization [27], [28], because premium prices are paid and product purchase is guaranteed. Moreover, trading NTFPs within a partnership was associated with higher levels of food consumption and less income inequality between women and men, both of which may be more important to the well-being of remote rural inhabitants. As our ethnographic data illustrated, men and women participated in NTFP gathering in all case studies, but when there was a partnership in place, product selling was guaranteed and the financial returns were superior because of higher than the average market price paid.

However, against our expectations, the results did not indicate that partnerships contribute to smoothing the typical fluctuations in forest income levels [13], [25], [73], such as proposed elsewhere [2]. Although there was a tendency to observe less irregular incomes when there was a company-community partnership, the result was not consistent across households. Probably, fluctuations in income across the year still occurred because several NTFPs are highly seasonal and companies purchased only one or two products, a typical situation of these company-community deals [74]. Partnerships may thus provide a safer outlet for NTFPs, but are also unable to stabilize income across the year. Furthermore, partnerships alone represented the commercialization strategy associated with the largest decline in leisure time, which we speculate might be a consequence of the tasks associated with dealing with companies and other third parties involved in trading. Other studies [47] have also reported overloaded daily commitments following the implementation of projects for trading NTFPs, even in the absence of partnerships. Because partnerships usually involve more managerial duties, negotiation, and higher levels of product control, they probably increase further people's time investments. Although we showed that the levels of food consumption were not affected, other aspects of the local livelihoods might have suffered. For instance, because our *leisure* variable included time dedicated to socialization and rituals, it was then likely that partnerships could be associated with negative impacts on local social capital (e.g., investing in social relations) and cultural activities. In fact, a previous study in one of the Caboclo communities in our sample (Roque) showed smaller time investments in social and communal activities when compared with another community without a partnership [34]. Despite that, we should acknowledge that the groups still had plenty of leisure time, and the percentages of reduction when compared with the baseline, although consistent, were relatively low (∼3%). Additionally, we cannot exclude that partnerships may have increased another type of social capital, i.e. external social capital, acquired through establishing links with companies, NGOs and other external players.

With respect to conservation, partnerships without product processing were associated with the second best result in terms of forest areas cleared and, thus, could be helping to curb deforestation, one of the main goals of the implementation of NTFP trade and partnerships with companies [29]. Conversely, partnerships were associated with the worst result in terms of wild animal offtake, so they are likely to have ambiguous results in terms of conservation. As previously argued, more access to monetary income is frequently associated with increased hunting harvests [75], [76] because people access guns and ammunition more easily [77]. Otherwise, people may reduce hunting if they substitute wild meat for other protein sources, such as purchased food or fish. In our context, however, communities live in remote locations where access to purchased protein is infrequent and expensive, unless people purchase meat locally. Fish is largely available, but perhaps, wild meat is considered a superior good in economic terms, so people are likely to increase the consumption with higher levels of income rather than reducing them [68]. Note also that NTFP gathering is frequently combined with hunting in tropical forests, as the association decreases the opportunity costs of hunting, because more time is spent in the forest environment [78]. By increasing income levels, partnerships may therefore be associated with decreases in the biomass of hunted animals, especially medium- to large-sized mammals [79], which in the long run could have cascading effects that would possibly impact the very NTFPs that are traded [80].

Results associated with the combination of product processing and a partnership were similar as regards economic indicators to those where there was only a partnership. But this combination performed slightly better in relation to leisure, deforestation and hunting impact. The most noteworthy aspect is that, against our expectations, the combination of product processing and a partnership had better economic indicators when compared to the baseline, but did not perform better than a partnership alone. We initially hypothesized that the combination would perform better in terms of income and income distribution, because it could expand income-earning opportunities created by product processing [47], while guaranteeing product purchasing within trade partnerships [29]. Moreover, product processing would contribute to reduce income fluctuations, but we observed neither of those effects.

Our last and most controversial finding relates to the worst-case scenario: the implementation of NTFP processing without the presence of a partnership. In this case, proxies of well-being displayed mostly negative results for households adopting this strategy. Recall that, compared to the baseline, total income, NTFP income, food consumption, and leisure were negatively correlated with a partnership, whereas income irregularity increased. Similarly, the area deforested was not affected, while hunting effort peaked. The evidence that the average total income and NTFP income were lower than when raw products were traded in commodity markets (i.e., the excluded category) is of particular interest, because it contradicts popular assumptions that product processing may add value to raw products [13], therefore enhancing the poor financial returns of NTFP trade [11], [14], and reducing fluctuations in income levels.

There are several plausible explanations for this finding. First, we cannot discard the possibility that the communities adopting this strategy in our sample were also those with less access to other income sources, which would explain their lower income levels; however, we also lack evidence to support this alternative argument. Moreover, this argument would not explain why families that adopt processing perform worst than the baseline in terms of NTFP income. A perhaps more plausible explanation is that product processing may add value to NTFP production [13], [25], though there are also opportunity costs incurred. Processing in the absence of partnerships may demand labor investments that are uncompensated by product selling, therefore diverting people from more profitable commercial activities or imperiling local subsistence production. Our ethnographic evidence and results from statistical analyses seem to support this view for two reasons. First, the observation that food consumption was lower in households adopting only NTFP processing lends credence to this explanation. Our ethnographic evidence also showed that some people, even in those communities where product processing was associated with partnerships, abandoned the processing phase after experimenting it for a year. People did so because they had difficulties in combining NTFP processing with agriculture, even when agricultural production was solely for the family consumption. As the preparation of agricultural plots was frequently contemporaneous with product processing, some households with fewer adults experienced food shortages, which were not compensated by supplementary monetary income derived from processing. Because of that, in subsequent years they participated only in gathering, but not in processing. Second, based on our preliminary hypotheses, if both product processing and partnerships were good, we should have observed the combination of the former to have displayed the best results; however, we found only intermediate results. The finding regarding product processing may seem controversial, but there is previous evidence that the local transformation of NTFPs may not necessarily be a good choice for forest communities in some contexts. For example, Marshall and colleagues [47] presented evidence that returns obtained from processed NTFPs do not compensate for the increased labor demand and costs incurred (taxes, legal). Similar evidence that processing is not necessarily profitable come from some cases of community managed timber in the Amazon [81], particularly in small, isolated operations with very small production volumes [82]. Therefore, although our finding could be contextual, it might also indicate a problem with projects aimed at adding value to NTFP production by processing, particularly in remote contexts where inhabitants must rely on their own production of food, and when processing interferes with other income sources and food production activities. Nonetheless, we should also highlight two other aspects. First, we have studied only remote communities and in this context the problems associated with spending time in processing may be exacerbated, because people can hardly substitute local food production with food from markets. Second, technological improvements in processing may perhaps increase productivity to a level that returns outweigh the costs.

Our results have several implications. First, when compared to the trade of NTFPs in commodity markets, establishing partnerships can enhance several outcomes of NTFP trade for local communities' well-being and conservation. Second, practitioners must use caution when promoting product processing because processing, at least in our context of remote forest communities, was associated with fewer benefits than merely gathering products to sell in commodity markets. Under certain conditions, processing may add value to NTFP production and even increase the monetary income of local people, but at the same time it can reduce total income (i.e., the sum of local production and monetary income) and the value of food consumption. Third, partnerships may reduce trade-offs between well-being and conservation in regards to deforestation, but two other trade-offs may persist. Improvements in well-being indicators observed in partnerships alone were still associated with the worst results in terms of more pervasive environmental impacts, such as wild animals offtake. Moreover, another less cited trade-off was also present. Improvements in standard economic attributes may be associated with declines in indicators of local socialization and cultural activities, implying that partnerships could be established at the expense of social and cultural capital or even financial capital, in case people stop relying on mutual support mechanisms and the transference of resources through gifts. At least for one of the communities studied (i.e., Roque), increased commercialization of processed NTFPs within a partnership was associated with a decrease in the amount of resources shared among households, which mainly consisted of food transfers [34].

### Supporting Information

Abstracts in Portuguese and Spanish are provided in Abstracts S1.

## Supporting Information

Abstracts S1 **Supplementary Abstracts in Portuguese and Spanish.**
(DOC)
