# Supplementary material for: The Effects of Processing Non-Timber Forest Products and Trade Partnerships on People's Well-Being and Forest Conservation in Amazonian Societies
Source: PLoS One. 2012 Aug 17;7(8):e43055. doi: 10.1371/journal.pone.0043055 (PMC3422238; doi:10.1371/journal.pone.0043055)
Supplement: Abstracts S1 — Supplementary Abstracts in Portuguese and Spanish. (DOC) [file pone.0043055.s001.doc]

**Abstract in Portuguese**

**Os efeitos das parcerias comerciais com empresas e do processamento de produtos florestais não-madeireiros no bem-estar humano e na conservação de florestas tropicais em sociedades amazônicas**

**Resumo**

Este estudo avaliou os efeitos do estabelecimento de parcerias comerciais com empresas e do processamento de produtos florestais não madeireiros (PFNM) como forma de maximizar os resultados positivos da comercialização. Especificamente, foi avaliada a associação de três estratégias de comercialização, quais sejam (i) o processamento de produtos florestais, (b) as parcerias comunidade-empresa e (iii) uma combinação de ambas estas estratégias, com vários indicadores de bem-estar e de conservação florestal. A análise foi baseada em dados etnográficos e quantitativos (i.e., *survey* e técnicas de observação direta), coletados em sete comunidades pertencentes a cinco sociedades da Amazônia brasileira e boliviana. Os resultados indicaram que o processamento de produtos e as parcerias nem sempre são uma solução ótima para alavancar os efeitos positivos da comercialização de PFNM no bem-estar e na conservação de florestas tropicais. Unidades domésticas que estabeleceram parcerias para a comercialização de PFNMs, mas não processaram os produtos florestais apresentaram melhores indicadores de bem-estar (renda total, renda de PFNMs, renda regular e consumo de alimentos) quando comparadas às unidades sem intervenções. A combinação de processamento de produtos com a existência de parcerias com empresas teve resultados semelhantes. Inesperadamente, o processamento de PFNMs em contextos de ausência de parcerias comerciais apresentou uma associação negativa com indicadores econômicos de bem-estar. Todas as estratégias de comercialização de PFNM investigadas mostraram estar associadas com uma redução no tempo dedicado a atividades sociais e culturais. No caso da conservação florestal, as estratégias que combinaram a parceria com ou sem processamento produziram resultados similares: enquanto a área desmatada por família tendeu a diminuir, o impacto da caça (medida pelo esforço de caça, ou consumo de animais selvagens) aumentou. O processamento sem parcerias está mais comumente associado à elevação da caça, mas não a reduções no desmatamento. Os resultados deste estudo indicam que o estabelecimento de parcerias para a comercialização de PFNM pode melhorar alguns dos indicadores econômicos locais, mas o processamento de PFNM pode ter impactos negativos nas atividades sociais e culturais. Em matéria de conservação, as três estratégias estudadas parecem reduzir o desmatamento, muito embora outros impactos como a caça permaneçam.

**Palavras-chave:** produtos florestais não madeireiros; parcerias comerciais; processamento de produtos; conservação florestal; bem-estar; Amazonia.

**Abstract in Spanish**

**Los efectos de las alianzas con empresas y del procesamiento de productos forestales no maderables en el bienestar humano y la conservación forestal en sociedades Amazónicas**

**Resumen**

Este estudio evaluó los efectos del procesamiento de productos forestales no maderables (PFNM) y del establecimiento de alianzas comerciales entre comunidades forestales y empresas, en la maximización de los beneficios de su comercialización. Específicamente, evaluamos la asociación de tres estrategias comerciales (i.e., (i) procesamiento de productos, (ii) las alianzas comerciales, o (iii) una combinación de ambas estrategias) con varios indicadores de bienestar y con la conservación de los bosques tropicales. El análisis se basó en datos etnográficos y cuantitativos (i.e., encuestas y observaciones sistemáticas) recogidos en siete comunidades de cinco sociedades de la Amazonia brasileña y boliviana. Nuestros resultados indicaron que procesar productos y establecer alianzas no es siempre la mejor solución para mejorar los efectos de la comercialización de PFNM en el bienestar y la conservación. Los hogares que establecieron alianzas para la comercialización de PFNM pero no procesaban los productos, disfrutaban de mejores indicadores de bienestar (ingreso total, ingreso por PFNM, ingresos regulares, consumo de comida, e igualdad en la repartición del ingreso entre mujeres y hombres) que los hogares que no habían establecido ningún tipo de alianza. La combinación del procesamiento de productos y alianzas comerciales presentó resultados similares. Inesperadamente, el procesamiento de PFNM sin alianzas comerciales presentó una asociación negativa con los indicadores de bienestar. Todas las estrategias relacionadas con la comercialización de PFNM estaban asociadas con una reducción en el tiempo dedicado a actividades sociales y culturales. Las estrategias que incluían alianzas comerciales con o sin procesamiento produjeron resultados similares respecto a la conservación: la superficie deforestada por hogar disminuyó mientras que el impacto de la caza (medido por el esfuerzo de caza y el consumo de animales silvestres) aumentó. Los resultados de este artículo indican que el establecimiento de alianzas para la comercialización de PFNM puede conllevar a la mejora de algunos de los indicadores económicos. Sin embargo, el procesamiento de PFNM sin alianzas puede tener impactos negativos en actividades sociales y culturales. Respecto a la conservación, las tres estrategias estudiadas parecen reducir la deforestación, aunque pueden incrementar otros impactos como la caza.

**Palabras clave:** productos forestales no maderables; alianzas comerciales; procesamiento de productos; conservación; bienestar; Amazonia
